# Statin Use Is Associated With a Lower Risk of Blepharitis: A Population-Based Study

**DOI:** 10.3389/fmed.2022.820119

**Published:** 2022-03-15

**Authors:** Kathy Ming Feng, Chi-Hsiang Chung, Yi-Hao Chen, Wu-Chien Chien, Ke-Hung Chien

**Affiliations:** ^1^Department of Ophthalmology, Tri-Service General Hospital, National Defense Medical Center, Taipei, Taiwan; ^2^Department of Medical Research, Tri-Service General Hospital, National Defense Medical Center, Taipei, Taiwan; ^3^School of Public Health, National Defense Medical Center, Taipei, Taiwan; ^4^Taiwanese Injury Prevention and Safety Promotion Association, Taipei, Taiwan; ^5^Graduate Institute of Life Sciences, National Defense Medical Center, Taipei, Taiwan

**Keywords:** statin, blepharitis, meibomian gland dysfunction (MGD), cohort-study, HMG-CoA

## Abstract

**Background:**

Blepharitis is a common eye disorder that may be overlooked by patients and clinical practitioners. The symptoms of blepharitis often manifest as irritation, a burning sensation, grittiness, and itchiness and may decrease visual acuity if not treated promptly. Meibomian gland dysfunction (MGD), a common cause of blepharitis, is believed to be associated with increased inflammatory marker levels that may disrupt the composition of lipids produced by the sebaceous glands in the eyelids and ultimately cause tear film instability.

**Methods:**

This is a retrospective, population-based study using National Health Insurance Research Database (NHIRD) data from a 14-year period (2000–2015). Pearson chi-squared and Student's *t*-tests were used to assess the differences in categorical and continuous variables, respectively, between statin users and non-statin users. Univariate and multivariate Cox regression analyses were performed to calculate the hazard ratios (HRs) after adjusting for confounders. Kaplan-Meier analysis was used to assess the cumulative risk of blepharitis between the two cohorts.

**Results:**

A total of 67,014 patients who used statins were enrolled as the study cohort, and 268,056 patients who did not use statins were enrolled as the comparison cohort. The incidence of blepharitis was 3.04% with statin treatment and 3.72% without statin treatment (*p* < 0.001). Patients who used statins had a lower risk of developing blepharitis [adjusted hazard ratio (aHR): 0.746, *p* < 0.001] than those who did not. In addition, diabetes mellitus (DM), hypertension, coronary heart disease (CHD), stroke, chalazion, rosacea, Sjogren syndrome, psoriasis and atopy were found to be possible risk factors for blepharitis.

**Conclusion:**

Statin use can decrease the risk of developing blepharitis. However, further prospective studies are needed to evaluate statin treatment for various subtypes of blepharitis and to identify the associated mechanism.

## Introduction

Blepharitis, which is commonly observed in clinical settings, is an eye condition that affects individuals in all age groups. It is characterized by irritation, itchiness and tear film instability, which ultimately lead to ocular surface inflammation (1). There are few epidemiological data on blepharitis; however, US ophthalmologists and optometrists reported that 37–47% of patients had signs of blepharitis in their survey (2). The pathogenesis of blepharitis is multifactorial; bacteria and MGD are strongly associated with it (3, 4), and it can be classified according to the length of the disease process and its anatomic location. Anterior blepharitis is characterized by staphylococcal and seborrheic blepharitis, and posterior blepharitis affects the posterior lid margin. Staphylococcal bacteria on the ocular surface are believed to play a vital role in the development of staphylococcal blepharitis, and hordeola are often associated with it (2, 4). Posterior blepharitis is more often associated with meibomian gland dysfunction (MGD) and other etiologies, such as allergic conjunctivitis and systemic conditions. This form is often characterized by telangiectasia of the lid margin, pouting and plugging of meibomian gland orifices, secretions of turbid to thick, opaque meibum, and thickened and irregular lid margins (5). This results in an altered composition of meibum, instability of tear film, atrophy of meibomian glands, and increased bacterial growth, all of which ultimately cause ocular surface inflammation. Patients often present with irritation, a burning sensation, itchiness, grittiness and crusting and redness of the eyelid margins. Lipid production by meibomian glands or other sebaceous glands and its composition are essential for tear film stability (4, 5).

The discovery of statins in the 1970s was a breakthrough for the prevention of hypercholesteremia. Their antiatherosclerotic effects significantly reduce the incidence of many coronary events. Statins, or 3-hydroxy-3-methylglutaryl coenzyme A (HMG-CoA) reductase inhibitor, bind to HMG-CoA and alter the conformation of this enzyme, which precludes the conversion of HMG-CoA into the cholesterol precursor mevalonic acid (6). The inhibition of this process leads to many pleiotropic effects, and the anti-inflammatory effect has been increasingly recognized (7).

In patients with blepharitis and MGD, levels of metalloproteinase (MMP)-9, interleukin (IL)-1β, IL-6, IL-17, tumor necrosis factor (TNF)-α and intercellular cell adhesion molecule-1 (ICAM-1) were found to be upregulated in tear fluid, and these inflammatory markers amplify the inflammatory response, disrupt tight junctions of the corneal epithelium, and induce goblet cell apoptosis, which ultimately disrupt the stability of the tear film (8, 9). Interestingly, HMG-CoA expression was found in all sebaceous glands in human eyelid tissues, and a recent pilot study reported a reduction in blepharitis symptoms and signs with topical statin treatment (10, 11). This raises the possibility that statins are able to directly affect lipid production in the eyelid and may decrease blepharitis risk. However, a prospective study in Taiwan found that statin use was unable to reverse lid margin meiboscores and meibum quality in patients with blepharitis, and the Blue Mountains Eye Study III showed the presence of dry eye symptoms with statin usage (12, 13). However, a large, clinical study evaluating the role of statins in the prevention of blepharitis is lacking. The purpose of this study was to evaluate the impact of oral statin use on the risk of developing blepharitis in a Taiwanese population and to identify the potential risk factors for blepharitis.

## Materials and Methods

### Data Source

The outpatient data were obtained from the Longitudinal Health Insurance Database (LHID) of the National Health Insurance Research Database (NHIRD), which is managed by the National Health Research Institute. The National Health Insurance program covers more than 99% of the population in Taiwan. This study was conducted from 1 January 2000 to 31 December 2015 (a 14-year period). Patient demographics (sex, age, index year and related comorbidities) were recorded. All medical diagnoses were determined according to the International Classification of Diseases, Ninth Revision, Clinical Modification (ICD-9-CM). This study was conducted according to the Code of Ethics of the World Medical Association (Declaration of Helsinki) and approved by the Institutional Review Board of Tri-Service General Hospital (TSGHIRB No. B-110-50); the need for informed consent was waived because fully anonymized data from the NHIRD were used.

### Study Design and Participants

A retrospective matched-cohort study was conducted on patients who first received statin therapy between 2000 and 2015. Patients 18 years of age and older who received statin treatment at least three times during outpatient visits were included. The exclusion criteria were as follows: treatment with statins before 2000, blepharitis before statin treatment, age younger than 18 years, unknown sex and loss to follow-up. Propensity score matching was applied at a 1:4 ratio to construct the comparison cohort. These randomly selected patients without statin use were matched with patients with statin use according to sex, age, and index year following the same exclusion criteria. The tracking endpoint was defined as the date of newly developed blepharitis or the end of the study period. Blepharitis was identified by the ICD-9-CM code 373x.

### Covariates

The comorbidities that were evaluated at baseline were as follows: diabetes mellitus (DM), hypertension, depression, anxiety, hyperthyroidism, coronary heart disease (CHD), stroke, chalazion, rosacea, Sjögren syndrome, psoriasis and atopy. In addition, the Charlson comorbidity index revised (CCI_R) was used to assess the presence of chronic diseases.

### Statistical Analysis

To evaluate the difference between the statin treatment group and the group without statin treatment, categorical variables were analyzed using the Pearson chi-square test and Fisher's exact test and are expressed as numbers and percentages. Continuous variables were compared using Student's *t*-test and are expressed as the means ± standard deviations. Multivariable Cox proportional hazards regression analysis was used to assess the risk of blepharitis, and the results are reported as adjusted hazard ratios (aHRs) with 95% confidence intervals (CIs). Survival analysis using the Kaplan-Meier method with a log-rank test was used to assess the outcome of blepharitis development between the two cohorts. A two-tailed *p* value < 0.05 was considered indicative of statistical significance. All analyses were performed using SPSS software version 22 (SPSS Inc., Chicago, Illinois, USA).

## Results

**Figure 1:**
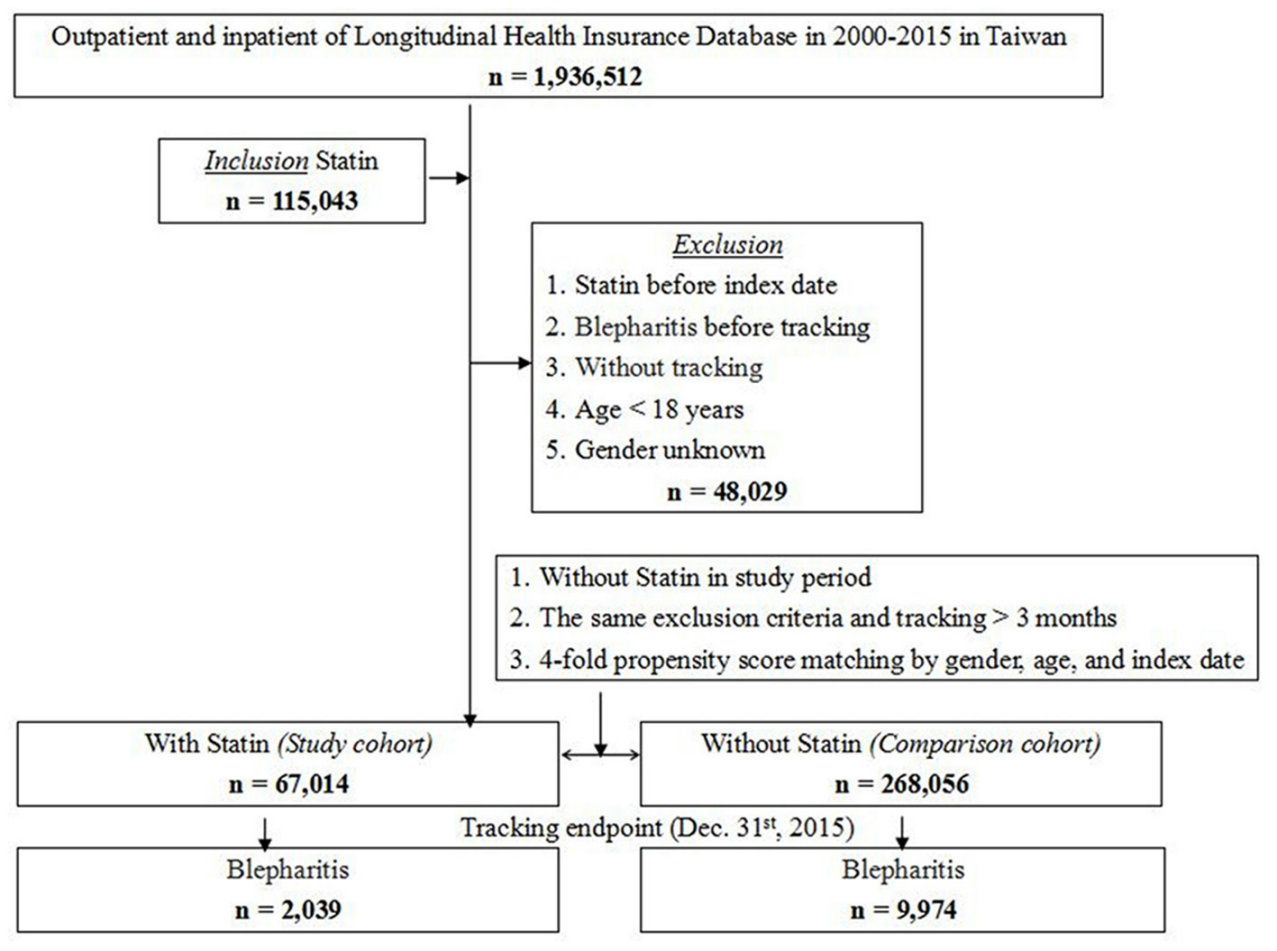
Flowchart of patient selection.

A total of 1,15,043 patients out of the 1,936,512 patients in the LHID received statin treatment during 2000–2015. Among these, 48,029 patients were excluded based on the exclusion criteria, and 67,014 patients were included in the study cohort. With 1:4 matching by sex, age and index date, 2,68,056 patients without statin treatment were selected as the control cohort. The study flowchart is illustrated in Figure 1.

### Patient Characteristics

The mean age at the tracking endpoint was higher among patients with statin treatment (61.02 ± 14.97 years) than among those without statin treatment (60.35 ± 14.58 years) (*p* < 0.001). As depicted in Table 1, the mean age at baseline was 54.26 ± 13.36 years and 54.20 ± 13.27 years for the study and control cohorts, respectively. The mean follow-up time was 12.84 ± 10.47 years for all patients (Supplementary Table S1-1). As shown in Supplementary Table S1-2, we found that patients treated with statin had a longer period to develop blepharitis (mean ± SD = 8.55 ± 3.82 years) than patients without statin treatment (mean ± SD = 8.23 ± 3.70 years) with *p* < 0.001. The incidence of newly developed blepharitis was significantly lower with statin use (3.04%) than without statin use (3.72%) (*p* < 0.001). In the study population, most patients were aged 60 years or older (32.79%). Among the comorbidities, DM, hypertension, depression, anxiety, CHD, stroke, rosacea, Sjögren syndrome, psoriasis and atopy had significantly higher incidences in patients taking statins than in those not taking statins (*p* < 0.001). Furthermore, the CCI_R score was slightly higher in the statin group (*p* < 0.001).

**Table 1:** Demographics characteristics at baseline (*n* = 3,35,070).

	**Statin**			
	**With (*****n*** **=** **67,014)**		**Without (*****n*** **=** **2,68,056)**		***p-*value**
	***n***	**%**	***n***	**%**	
**Gender**					0.999
Male	33,785	50.41	1,35,140	50.41	
Female	33,229	49.59	1,32,916	49.59	
**Age, mean (years)**	54.26 ± 13.36	54.20 ± 13.27	0.296
**Age groups (yrs)**					0.999
18–29	5,223	7.79	20,892	7.79	
30–39	9,786	14.60	39,144	14.60	
40–49	11,240	16.77	44,960	16.77	
50–59	18,794	28.04	75,176	28.04	
60	21,971	32.79	87,884	32.79	
**Pre-existing comorbidities**
**Diabetes mellitus**	18,875	28.17	39,801	14.85	<0.001^*^
**Hypertension**	17,985	26.84	38,701	14.44	<0.001^*^
**Depression**	5,119	7.64	12,450	4.64	<0.001^*^
**Anxiety**	6,635	9.90	14,010	5.23	<0.001^*^
**Hyperthyroidism**	1,010	1.51	2,214	0.83	<0.001^*^
**CHD**	6,684	9.97	22,097	8.24	<0.001^*^
**Stroke**	6,420	9.58	20,785	7.75	<0.001^*^
**Chalazion**	1,065	1.59	2,984	1.11	0.007^*^
**Rosacea**	2,771	4.13	4,801	1.79	<0.001^*^
**Sjögren syndrome**	4,098	6.12	10,126	3.78	<0.001^*^
**Psoriasis**	1,806	2.69	3,504	1.31	<0.001^*^
**Atopy**	1,645	2.45	3,287	1.23	<0.001^*^
**CCI_R**	0.06 ± 0.15	0.04 ± 0.09	<0.001^*^

*P: Chi-square/Fisher exact test on category variables and t-test on continue variables. CHD, Coronary Heart Disease; CCI-R, Charlson comorbidity index revised*.

* *Denotes statistically significant*.

### Outcomes

**Figure 2:**
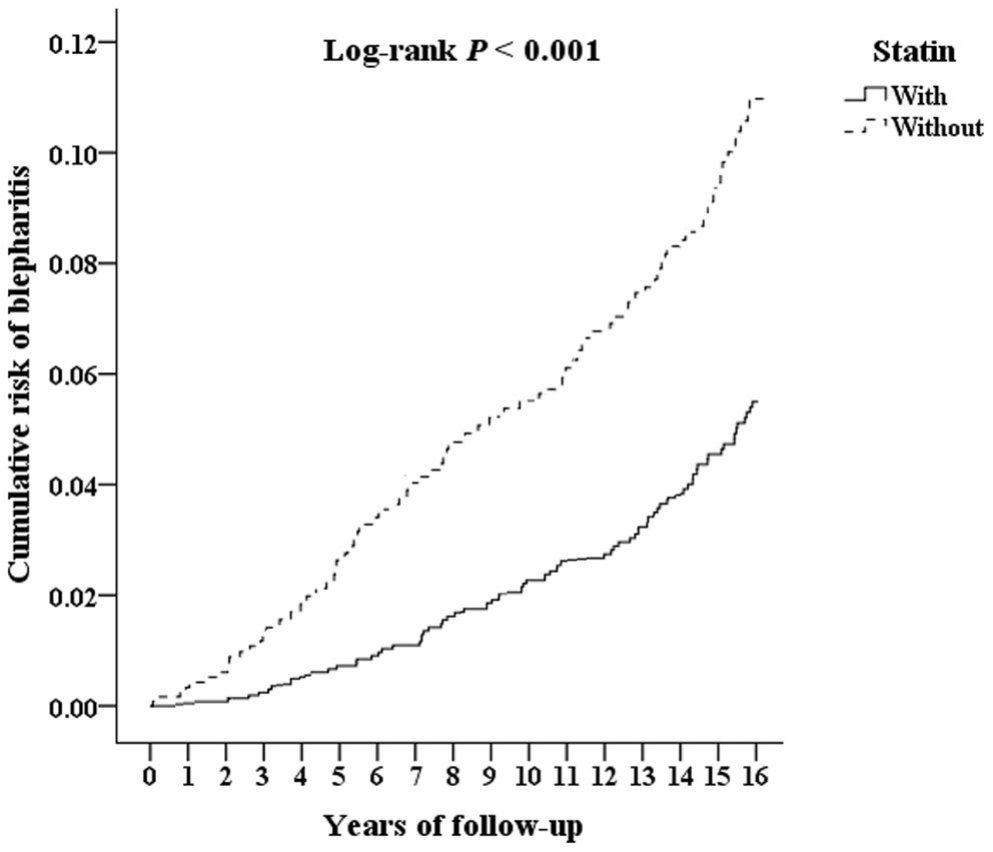
Kaplan-Meier survival curve for blepharitis in patients with statin and without statin use.

The Kaplan-Meier survival curve of the cumulative risk of blepharitis in patients with statin treatment was significantly lower than that in patients without statin use (*p* < 0.001), as shown in Figure 2. As shown in Table 2, after adjusting for age, sex and comorbidities, multivariate analysis using Cox regression revealed that patients who used statins had a significantly decreased risk for developing blepharitis compared to those who did not use statins (aHR = 0.746, *p* < 0.001). Patients aged 40 years and older showed an increased risk of developing blepharitis compared to those aged 18–29 years old. Patients with DM, hypertension, chalazion, rosacea, Sjögren syndrome, psoriasis, and atopy had a significantly increased risk of developing blepharitis compared to those without these comorbidities.

**Table 2:** Predictors of overall blepharitis by cox regression analysis.

	**Crude HR (95% CI)**	***P***	**Adjusted HR (95% CI)**	***P***
**Statin**	0.689 (0.467–0.872)	<0.001^*^	0.746 (0.528–0.931)	<0.001^*^
Male	0.989 (0.787–1.064)	0.301	1.030 (0.807–1.172)	0.255
**Age groups (yrs)**				
18–29	Reference		Reference	
30–39	1.024 (1.015–1.038)	0.036^*^	1.002 (0.985–1.024)	0.076
40–49	1.062 (1.048–1.080)	0.002^*^	1.042 (1.017–1.064)	0.033^*^
50–59	1.097 (1.064–1.155)	<0.001^*^	1.074 (1.040–1.085)	<0.001^*^
≧60	1.189 (1.104–1.197)	<0.001^*^	1.091 (1.079–1.131)	<0.001^*^
**Diabetes mellitus**	1.983 (1.531–2.688)	<0.001^*^	1.886 (1.400–2.593)	<0.001^*^
**Hypertension**	1.87 (1.283–2.537)	<0.001^*^	1.825 (1.231–2.286)	<0.001^*^
**Depression**	1.345 (1.092–1.518)	0.002^*^	1.266 (0.936–1.402)	0.083
**Anxiety**	1.424 (1.102–1.630)	<0.001^*^	1.271 (0.965–1.384)	0.081
**Hyperthyroidism**	1.518 (0.276–1.939)	0.870	1.472 (0.223–1.889)	0.706
**CHD**	1.526 (1.223–1.873)	<0.001^*^	1.480 (1.095–1.791)	<0.001^*^
**Stroke**	1.597 (1.245–1.986)	<0.001^*^	1.505 (1.193–1.806)	<0.001^*^
**Chalazion**	1.915 (1.429–2.207)	<0.001^*^	1.699 (1.320–2.002)	<0.001^*^
**Rosacea**	1.803 (1.442–2.183)	<0.001^*^	1.663 (1.262–1.981)	<0.001^*^
**Sjögren syndrome**	1.404 (1.297–1.620)	<0.001^*^	1.374 (1.251–1.588)	<0.001^*^
**Psoriasis**	1.599 (1.284–1.986)	<0.001^*^	1.583 (1.226–1.835)	<0.001^*^
**Atopy**	1.493 (1.157–1.882)	<0.001^*^	1.480 (1.131–1.702)	<0.001^*^
**CCI_R**	1.32 (1.205–1.455)	<0.001^*^	1.303 (1.195–1.380)	<0.001^*^
**Medical visits**	1.099 (1.057–1.130)	<0.001^*^	1.095 (1.051–1.126)	<0.001^*^

*HR, hazard ratio; CI, confidence interval; Adjusted HR, adjusted variables listed in table; CHD, coronary heart disease; CCI_R, charlson comorbidities index revised*.

* *Denotes statistically significant*.

Table 3 presents the stratified analyses comparing the risk of developing blepharitis between patients with and without statin use according to each evaluated variable. The risk of developing blepharitis was lower in patients with statin use than in those without statin use for both sexes and all age groups. Regardless of whether patients had comorbidities (DM, hypertension, depression, anxiety, hyperthyroidism, CHD, stroke, chalazion, rosacea, Sjögren syndrome, psoriasis and atopy), patients with statin use had a lower risk of developing blepharitis than those without statin treatment. At the study endpoint, 2,039 (3.04%) patients with statin use developed blepharitis, compared with 9,974 (3.72%) patients without statin use.

**Table 3:** Risk analysis for blepharitis stratified by demographic and clinical characteristics between patients with statin/without statin use.

	**With statin**		**Without statin**		**With vs. without (Reference)**
**Stratified**	**Events**	**Rate (per 10^**5**^ PYs)**	**Events**	**Rate (per 10^**5**^ PYs)**	**Adjusted HR (95% CI)**	***P***
**Overall**	2039	236.05	9974	290.01	0.746 (0.528–0.931)	<0.001^*^
**Gender**						
**Male**	1157	265.73	5551	320.03	0.768 (0.532–0.952)	<0.001^*^
**Female**	882	205.88	4423	259.47	0.683 (0.511–0.900)	<0.001^*^
**Age groups (yrs)**						
18–29	144	215.45	752	282.04	0.703 (0.492–0.871)	<0.001^*^
30–39	279	223.28	1430	285.97	0.718 (0.501–0.893)	<0.001^*^
40–49	310	230.09	1665	289.47	0.735 (0.522–0.917)	<0.001^*^
50–59	581	242.03	2901	300.83	0.747 (0.526–0.929)	<0.001^*^
≧60	725	243.91	3226	284.75	0.789 (0.552–0.980)	0.035^*^
**Diabetes mellitus**	459	188.60	1135	222.17	0.750 (0.542–0.938)	<0.001^*^
**Hypertension**	444	191.18	1101	221.02	0.751 (0.530–0.942)	<0.001^*^
**Depression**	183	276.80	545	340.82	0.748 (0.531–0.937)	<0.001^*^
**Anxiety**	217	252.52	555	308.14	0.766 (0.539–0.952)	0.001^*^
**Hyperthyroidism**	49	356.34	108	378.52	0.769 (0.536–0.978)	0.027^*^
**CHD**	139	158.82	448	151.02	0.777 (0.522–0.953)	0.007^*^
**Stroke**	153	181.99	489	176.11	0.794 (0.538–0.961)	0.009^*^
**Chalazion**	35	241.61	111	285.01	0.789 (0.559–0.978)	0.026^*^
**Rosacea**	87	242.48	182	293.31	0.761 (0.530–0.944)	<0.001^*^
**Sjögren syndrome**	129	240.28	375	289.13	0.765 (0.538–0.950)	0.001^*^
**Psoriasis**	60	249.32	134	293.21	0.782 (0.549–0.972)	0.020^*^
**Atopy**	52	239.84	121	285.70	0.779 (0.551–0.968)	0.018^*^

*PYs, Person-years; Adjusted HR, Adjusted Hazard ratio; Adjusted for the variables listed in Table 2; CI, confidence interval; CHD, coronary heart disease*.

* *Denotes statistically significant*.

## Discussion

This 14-year follow-up population-based study revealed that after adjusting for covariates, patients with statin treatment had a lower risk of developing blepharitis than matched patients without statin treatment. In addition, an age of 40 years and older, DM, hypertension, stroke, CHD, chalazion, rosacea, Sjögren syndrome, psoriasis, and atopy conferred a significantly higher risk of developing blepharitis. Similarly, Kaplan-Meier analysis revealed that patients with statin treatment had a significantly lower risk of blepharitis than those without statin treatment. After stratified analyses, patients with statin treatment showed a decreased risk of developing blepharitis across all comorbidities. To the best of our knowledge, this is the first large-sample cohort study investigating the association of oral statin treatment and blepharitis and evaluating possible risk factors for blepharitis in patients receiving statin treatment.

Statins have anti-inflammatory and immunomodulatory effects (14). A comprehensive review by Ooi et al. explicitly examined the therapeutic effects of statins in ocular diseases such as uveitis, thyroid eye disease, glaucoma, cataracts, proliferative vitreoretinopathy, diabetic retinopathy, macular degeneration and choroidal melanoma (14). Patients with posterior blepharitis have inflammation of the posterior lid margin involving the meibomian gland, and this form is often associated with several systemic diseases (1, 3). An observational study published in 2013 revealed that MGD was significantly associated with increased systemic total cholesterol and low-density lipoprotein (LDL) levels, and another population-based study reported an aHR of 1.145 for an increased risk of developing metabolic syndrome in patients with blepharitis (15, 16). Meibomian secretions of meibum are composed of a mixture of lipids, including wax esters, cholesteryl esters, cholesteryl esters of (O-acyl)-ω-hydroxy fatty acids, triacylglycerols, free fatty acids, cholesterol and small amounts of polar and non-polar lipids (17). MGD causes excess free cholesterols on the ocular surface, which disrupt the stability of the tear film, causing inflammation and increased tear evaporation and tear osmolarity (18, 19). In addition, MGD can increase levels of proinflammatory cytokines such as MMP-9, which infiltrate the ocular surface epithelium by degrading tight junctions (8, 20). A prospective pilot study revealed that topical atorvastatin drops were able to significantly improve tear breakup time, the blepharitis score and conjunctival injection (10). In this study, we found a significantly lower risk of developing blepharitis in patients treated with statins after adjusting for confounding factors. *In vitro* studies have demonstrated that atorvastatin downregulates T helper cells and thereby decreases inflammatory cytokines such as IL-1β, IL-6, IL-17 and IFN-γ (21), which are elevated in blepharitis and dry eye patients (8, 14). IFN-γ has been found to induce goblet cell apoptosis in the conjunctiva, whereas IL-1β, IL-6, IL-17 and TNFα have been shown to increase the MMP-9 concentration in keratocytes (14).

The exact mechanism between blepharitis, MGD, and statin use is not clear. A recent study revealed that HMG-CoA reductase, which is involved in lipid secretion, was expressed not only in the meibomian gland of human eyelid tissue but also in Zeis and pilosebaceous glands (11), suggesting that statins may have a direct effect on lipid production in eyelid tissues. However, a recent prospective, clinical study in Taiwan showed that changes in meiboscores, lid margin abnormality scores and the quality of meibum were not reversed with the use of statins in dyslipidemia patients at a follow-up of 12 months, and no significant changes in Ocular Surface Disease Index (OSDI) scores or other dry eye parameters were observed (13). This could be related to the small sample size and the limited follow-up duration. A cross-sectional study from the Blue Mountains Eye study found that usage of statins was correlated with one or more symptoms of dry eye disease (12). However, this result is limited, as the study looked at symptoms of dry eye and lacked clinical examination results. Our study provides a longitudinal assessment with the same initial time point of statin treatment for a 14-year follow-up duration.

A retrospective case-control study in Israel demonstrated that blepharitis is associated not only with psychological conditions but also with systemic diseases and other eye diseases (22), the latter two being consistent with our results. Among possible systemic diseases, DM, hypertension, CHD, and stroke all contributed to a higher risk of developing blepharitis (*p* < 0.0010) in this study. Patients with a long-term DM diagnosis are more susceptible to infection due to reduced blood flow at the eyelid. Kruse et al. showed that in diabetic patients, the adjusted odds ratio for acquiring infectious conjunctivitis is 1.24 (23). Studies have also shown that diabetes can cause a reduction in goblet cell density in the conjunctiva and a poor microvascular supply to the lacrimal gland, resulting in tear film instability and blepharitis (24). Hence, patients with DM may have both anterior and posterior blepharitis occurring simultaneously, and our study showed that DM is a risk factor for blepharitis (aHR: 1.888, *p* < 0.001). However, the use of statin was able to decrease the risk of blepharitis in patients with DM (aHR:0.757, *p* = 0.001). In a population study assessing blepharitis as a potential indicator for metabolic syndrome, hypertension and DM were not correlated with blepharitis (16). However, a study by Nemet et al. and another population-based study reported a significant association of cardiovascular disease, including hypertension and coronary heart disease, with blepharitis (22, 25), which is consistent with our results. In addition, the International Workshop on Meibomian Gland Dysfunction also suggested hypertension as a risk factor for blepharitis (5). In general, eyelids are rich in vascular supply (26), and cardiovascular diseases may hinder the blood supply, inducing MGD and subsequently, blepharitis. Thus, it is important to recognize the systemic diseases associated with blepharitis early in order to implement treatment for these patients.

In this study, we found that hyperthyroidism was not associated with an increased risk of blepharitis (*p* < 0.711). However, thyroid eye disease (TED) is a common ocular manifestation in hyperthyroidism patients, where orbital fibroblasts are the main target of both humoral and cellular immunity (27). Patients with TED may experience lid retraction, exophthalmos, lagophthalmos, and impaired lacrimal gland secretion, and this condition ultimately leads to a poor ocular surface and becomes a risk factor for blepharitis (28). Active TED eyes showed significantly loss of meibomian glands than inactive TED (28). On another hand, Nemet et al. showed that hypothyroidism (OR: 1.2) was significantly associated with blepharitis as well, and another study reported an OR of 1.05, suggesting that hormonal levels can alter meibomian secretions (22, 29, 30). Hypothyroidism can result in an increase in cholesterol and LDL levels (31). T_3_ hormone can reduce plasma lipoprotein levels (32), and a low level of T_3_ hormone may cause an accumulation of lipids in the meibomian gland. Furthermore, the meibomian gland is the target tissue for androgen, estrogen and progesterone, and a reduction in these hormones is correlated with hypothyroidism (33). Thus, both hypothyroidism and hyperthyroidism are associated with blepharitis via different mechanisms. Since there was no specific code for thyroid eye disease, it was difficult to interpret the nature of TED in these patients with hyperthyroidism. Although we did not find an association of hyperthyroidism with blepharitis, statin use decreased the risk of blepharitis in patients with hyperthyroidism (aHR: 0.766, *p* = 0.023), suggesting that statin use can potentially alter lipid conditions on the ocular surface, thereby relieving symptoms of blepharitis.

Regarding psychological conditions, a population-based study in Taiwan revealed a higher risk of depression (aHR: 1.42) and anxiety (aHR: 1.57) in blepharitis patients (25). Likewise, Nemet et al. reported strong associations of anxiety (OR: 1.6) and depression (OR: 1.2) with blepharitis (22). Studies have reported that levels of chronic inflammatory cytokines such as IL-1β, IL-6, TNF-α and IFN-γ are higher in depressed patients (34), suggesting a similarity in the mechanism of blepharitis development. Interestingly, antidepressant use has a relative risk of 1.44 in the development of dry eye (35) due to the possibility of decreased lacrimal secretion. In this study, we observed an increased risk of blepharitis in depression and anxiety patients with aHRs of 1.265 and 1.274, respectively; however, the difference did not reach statistical significance. Nevertheless, under statin treatment, patients with depression and anxiety both have a lower risk of developing blepharitis (aHR: 0.756 and 0.761, respectively).

It is well known from the International Workshop of Meibomian Gland Dysfunction that aging, Sjögren's syndrome, psoriasis, atopy and rosacea may promote meibomian gland dysfunction, causing blepharitis (5), and our results support this. In fact, a population-based study in Korea also reported similar results with an increased risk of blepharitis in Sjögren's syndrome (HR: 1.64), psoriasis (HR: 1.11), atopy (HR: 1.35) and rosacea (HR: 1.53) (29). Aging is another risk factor for blepharitis (1). Lid margin morphology changes as we age. They become thicker, more hyperkeratinization and more telangiectasia which may ultimately increase the risk of blepharitis (36). The incidence of blepharitis was high in the elderly (>50 years) (29) and our study shows that the aHR for blepharitis also increases with age. Elderly may be associated with lower levels of androgen in men and hormonal changes associated with inflammatory cytokines in menopausal woman (37). Statin usage was able to decrease the risk of blepharitis in patients with all ages, chalazion, Sjögren's syndrome, psoriasis, atopy and rosacea. Anti-inflammatory effects of statin may have contribute to the decrease risk of blepharitis. There is a possibility that other confounding demographics factors and clinical characteristics could affect the association found in this study; however, multiple important confounding factors have been adjusted for in this analysis.

The major strength of our study is the large sample size and the long follow-up period, which provided good statistical power and reduced selection bias; hence, our study better reflected real-world situations than hospital-based studies. The control group was matched at a ratio of 1:4 at baseline along with possible adjustments for confounding factors using multivariate Cox regression analysis. However, there are several limitations to our study. First, this study was retrospective in nature, and we were unable to assess the lipid profile status at the time of statin use or the reason behind statin use. Patients with HTN, DM and CHD have separately been shown to develop blepharitis more commonly than their healthy counterparts, the use of statins in these patients may be associated with seeking medical care, compliance to medication, and control of the systemic condition. Thus, lower rates of blepharitis are detected may not solely due to the statin use, but due to the control of systemic condition. when compared to their non-compliant, untreated, or uncontrolled counterparts. We further analyze the medical visits of both group and found that in statin treatment group and without statin treatment group, the number of NHI claims for ophthalmic outpatient visits was 2.35 ± 3.04 and 2.33 ± 3.01, respectively (*p* = 0.125) and for overall medical visits was 13.34 ± 13.89 and 13.17 ± 13.62, respectively (*p* = 0.004) depicted in Supplementary Table S2. After adjustment for all medical visits, statin treatment was nonetheless associated with decrease risk of developing blepharitis (aHR: 0.746, *p* < 0.001). Second, other influential factors, such as other medications and the environment, that may contribute to the development of blepharitis could not be accounted for. Third, misclassification may have been possible, and we were unable to assess the subtypes of blepharitis (anterior or posterior).

## Conclusion

In conclusion, this retrospective, population-based cohort study demonstrated that patients who used statins had a lower risk of developing blepharitis than those who did not use statins. Furthermore, DM, hypertension, CHD, stroke, rosacea, Sjögren's syndrome, psoriasis, and atopy were associated with a higher risk of blepharitis development; however, statin use can decrease the risk of blepharitis in these patients and patients with depression or anxiety. Clinicians should be aware of the risks of blepharitis in patients with these underlying comorbidities. In the future, large well-designed clinical trials are required to confirm the association of statin use and blepharitis risk.

## Data Availability Statement

The original contributions presented in the study are included in the article/Supplementary Material, further inquiries can be directed to the corresponding authors.

## Ethics Statement

The studies involving human participants were reviewed and approved by Institutional Review Board of Tri-Service General Hospital (TSGHIRB No. B-110-50). Written informed consent for participation was not required for this study in accordance with the national legislation and the institutional requirements.

## Author Contributions

KF, W-CC, and K-HC: study design and manuscript writing. C-HC, Y-HC, and W-CC: data extracting and statistical analysis. KF, C-HC, Y-HC, W-CC, and K-HC: data checking. All authors contributed to the article and approved submitted version.

## Funding

This study was funded by the Tri-Service General Hospital Research Foundation (TSGH-D-110111 and TSGH-B-111018), and the sponsors have no role in study design, data collection and analysis, decision to publish, or preparation of the manuscript.

## Conflict of Interest

The authors declare that the research was conducted in the absence of any commercial or financial relationships that could be construed as a potential conflict of interest.

## Publisher's Note

All claims expressed in this article are solely those of the authors and do not necessarily represent those of their affiliated organizations, or those of the publisher, the editors and the reviewers. Any product that may be evaluated in this article, or claim that may be made by its manufacturer, is not guaranteed or endorsed by the publisher.
