# Supplementary material for: Statin Use Is Associated With a Lower Risk of Blepharitis: A Population-Based Study
Source: Front Med (Lausanne). 2022 Mar 15;9:820119. doi: 10.3389/fmed.2022.820119 (PMC8965040; doi:10.3389/fmed.2022.820119)
Supplement: Supplementary file 1 [file Table_1.DOCX]

**Supplementary Material**

S1-1. Years of follow-up

| **Statin** | **Min** | **Median** | **Max** | **mean ± SD** | ***p* Value** |
| --- | --- | --- | --- | --- | --- |
| Overall | 0.25 | 10.75 | 16.00 | 12.84 ± 10.47 | 0.185 |
| With | 0.25 | 10.97 | 16.00 | 12.89 ± 10.59 |  |
| Without | 0.25 | 10.61 | 16.00 | 12.83 ± 10.44 |  |

S1-2. Years to blepharitis

| **Statin** | **Min** | **Median** | **Max** | **mean ± SD** | ***p* Value** |
| --- | --- | --- | --- | --- | --- |
| Overall | 0.26 | 6.13 | 15.68 | 8.29 ± 3.73 | <0.001 |
| With | 0.26 | 6.33 | 15.68 | 8.55 ± 3.82 |  |
| Without | 0.26 | 5.82 | 15.62 | 8.23 ± 3.70 |  |

S2. Medical outpatient visits.

|  | **Statin** | |  |
| --- | --- | --- | --- |
|  | **With** | **Without** | ***p* Value** |
| **Medical visits (per year)** |  |  |  |
| Number of NHI claims for all outpatient visits during follow-up | 13.34 ± 13.89 | 13.17 ± 13.62 | 0.004 |
| Number of NHI claims for ophthalmic  outpatient visits during follow-up | 2.35 ± 3.04 | 2.33 ± 3.01 | 0.125 |
